# Higher intraoperative mean arterial blood pressure does not reduce postoperative delirium in elderly patients following gastrointestinal surgery: A prospective randomized controlled trial

**DOI:** 10.1371/journal.pone.0278827

**Published:** 2022-12-22

**Authors:** Yanke Zhang, Ying Zhang, Zhou Zhou, Xiaoqiao Sang, Miaomiao Qin, Guangrong Dai, Zhibin Zhao, Fang Yan, Xiaobao Zhang

**Affiliations:** 1 Department of Anesthesiology, The Affiliated Lianyungang Hospital of Xuzhou Medical University, Lianyungang, China; 2 Department of Basic Medical Science, Kangda College of Nanjing Medical University, Lianyungang, China; 3 Department of Anesthesiology, Kangda College of Nanjing Medical University, Lianyungang, China; University of Texas Medical Branch at Galveston, UNITED STATES

## Abstract

**Background:**

This study aimed to describe the relationship between the different levels of intraoperative mean arterial blood pressure (MAP) and postoperative delirium in elderly patients undergoing gastrointestinal laparoscopic surgery.

**Materials and methods:**

This prospective controlled clinical trial enrolled 116 patients aged 65 to 85 years who underwent gastrointestinal laparoscopic surgery. These patients were randomized 1:1 to a MAP goal of 65 to 85 mmHg (L group) or an 86 to 100 mmHg (H group). The primary endpoint was the incidence of postoperative delirium, assessed twice daily with the Confusion Assessment Method (CAM) and Richmond Agitation–Sedation Scale (RASS) during the first five postoperative days. Delirium severity was evaluated with the Delirium-O-Meter (D-O-M).

**Results:**

108 patients (L group n = 55, H group n = 53) were eventually included in intention-to-treat analyses. Postoperative delirium occurred in 18 (32.7%) of 55 cases of L group and in 15 (28.3%) of 53 cases of H group. The incidence of delirium subtypes between the two groups: hypoactive delirium 14.5% (8/55) vs 11.3% (6/53); hyperactive delirium 7.3% (4/55) vs 3.8% (2/53); mixed delirium 10.9% (6/55) vs 13.2% (7/53). However, the L group showed higher D-O-M scores of the first episode of delirium: 14.5 (Q1 = 12, Q3 = 18.5) vs 12 (Q1 = 10, Q3 = 14), which means the delirium is more severe.

**Conclusions:**

Compared with 65 to 85 mmHg, maintaining intraoperative MAP at 86–100 mmHg did not reduce the incidence of postoperative delirium in elderly patients undergoing gastrointestinal laparoscopic surgery. However, the severity of delirium could be reduced and blood loss is a risk factor for postoperative delirium.

## Introduction

Postoperative delirium is an acute onset and rapidly fluctuating mental disorder after surgery, accompanied by attention disorders and changes in the level of consciousness. The International Statistical Classification of Diseases and Related Health Problems 10th Revision (ICD-10) defines it as a brain syndrome with no specific organ attribution. Age is one of the risk factors for delirium. It mainly occurs in hospitalized patients older than 65 years, and the older the patient, the higher the incidence [1, 2]. One study showed that the incidence of delirium after cardiac surgery in elderly patients (≥ 65 years) was 21.4% and in very elderly patients (≥ 80 years) was 33.5% [3]. The occurrence of delirium is also closely related to the type of surgery. Hip fracture surgery has the highest reported incidence of delirium [4]. In addition, abdominal aortic aneurysm surgery, abdominal surgery, coronary artery bypass graft surgery and peripheral vascular surgery are also widely reported as types of surgery prone to delirium. Delirium can indicate a higher incidence of postoperative complications and they promote and influence each other, which means longer hospital stay and even more deaths [5].

Despite extensive research and literature on delirium, there is no efficient treatment yet [6]. Some researchers have maintained that low fluctuant intraoperative hemodynamic management is one of the risk-reducing interventions [7]. The general point here is that hypotension is a risk factor for delirium and low-level blood pressure management increase the incidence of postoperative delirium [8, 9]. However, some others studies also suggested that intraoperative hypotension does not correlate with delirium, and even the increased vasopressor intake to maintain a high level of MAP (≥90% of preoperative values) represents greater cardiovascular damage [10, 11]. The relationship between hemodynamics and delirium needs to be explored further.

## Materials and methods

### Study population

After obtaining approval from the Institutional Review Board (the Institutional Review Board of The Affiliated Lianyungang Hospital of Xuzhou Medical University, Jiangsu, China, Reference No: KY20200328002, Date of Approval: March 28, 2020), this study was registered at Chinese Clinical Trial Registry (Registration number: ChiCTR2000031657, Reg Date: April 6, 2020 http://www.chictr.org.cn). The authors confirm that all ongoing and related trials for this drug/intervention are registered. Patients were enrolled in the study if all the following criterions were met: (1)undergo gastrointestinal laparoscopic surgery and unplanned to be admitted to ICU; (2) between 65 and 85 years of age, (3) 18kg/m^2^ ≤ BMI ≤ 30kg/m^2^. Patients were excluded if any of the following was present: (1) with history of cerebrovascular accident; (2) systolic blood pressure exceeds 150 mmHg or diastolic blood pressure exceeds 90 mmHg; (3) with history of taking psychotropic drugs within half a year before hospitalization; (4) diagnosed with schizophrenia, epilepsy or Alzheimer’s disease; (5) neck ultrasound shows plaques in the blood vessels; (6) with visual, auditory, or language communication impairment and inability to complete delirium assessment; (7) with history of drug or alcohol abuse within 1 year, (8) had emergency surgery; (9) with a score of Mini-mental State Examination (MMSE) lower than the minimum score for the corresponding education level (illiteracy < 17, primary school < 20, secondary school < 22, university school < 23). After informed consent was obtained, the patients were allocated 1:1 to the L group, which maintains MAP in the range of 65 to 85 mmHg during the surgery, or the H group, which maintains MAP in the range of 86 to 100 mmHg during the surgery; this was done by a computer-generated block randomization. Patient were removed from the trial if any of the following occurs: (1) the duration of meeting the MAP target was less than 80% of the operative duration, (2) the operative duration was less than 1 h or more than 4 h, (3) unplanned admission to the ICU.

### Anesthetic regimen

All anesthetic techniques were standardized. Patients were fasting 8 h and abstained from drinking for 4 h before surgery. Noninvasive blood pressure, electrocardiogram and pulse oxygen saturation were routinely monitored after admission to the operating room. After establishing venous access, radial artery catheterization was performed to monitor arterial blood pressure. The induction of anesthesia was accomplished by intravenous administration of 0.3–0.5 μg/kg sufentanil, 1–2 mg/kg propofol, and 2 mg/kg cis-atracurium. The following were also administered intravenously: 0.1–0.3 μg/(kg·min) remifentanil, 4–8 mg/(kg·h) propofol, and 0.1–0.2 mg/(kg·h) cis-atracurium to maintain an appropriate anesthesia depth, which was monitored by bispectral index (BIS) to achieve a BIS value of 40–60. All patients were ventilated in volume-controlled mode with a 60% fractional concentration of inspired oxygen and tidal volume and respiratory rate were adjusted to achieve the partial pressure of end-tidal carbon dioxide fluctuating between 35 and 45 mmHg. Intraoperative blood pressure was controlled within the target range from the following three aspects: blood volume, heart rate, and vasoactive drugs. Fluid and blood products were replenished to maintain adequate circulation volume based on the duration of fasting and intraoperative blood loss. Heart rate was raised with atropine and lowered with esmolol based on the clinical experience of the anesthesiologist. If necessary, norepinephrine and urapidil were used to keep MAP in line with the blood pressure goals.

### Measurements and delirium evaluation

The primary endpoint was the incidence of postoperative delirium during the first 5 postoperative days, which was measured twice daily (6:00–8:00 and 16:00–18:00) by a trained researcher using CAM. CAM has always been the most widely used instrument to diagnose delirium since it was published in 1990 by Professor Inouye [12] because of its high sensitivity and specificity. CAM-CR is the Chinese reversion of CAM and it consists of 11 items with each item score ranging from 0(absent) to 4(severe) and the maximum possible score is 44 (scores > 22 is indicative of delirium). Delirium subtypes were measured jointly using CAM and RASS [13] (hypoactive delirium = CAM score > 22 and RASS score of 0 to -3; hyperactive delirium = CAM score > 22 and RASS score of 1 to 4; mixed delirium = fluctuates between hypoactive delirium and hyperactive delirium). Delirium severity was evaluated with D-O-M, which consists of 12 items (sustained attention, shifting attention, orientation, consciousness, apathy, psychomotor retardation/hypokinesia, incoherence, fluctuating, restlessness, delusions, hallucination, and anxiety) with each item was rated on a scale of 0, no pathology; 1, mild disturbances; 2, moderate; or 3,severe.

The duration of anesthesia (from the induction of anesthesia to the withdrawal of anesthetics), duration of operation (from skin incision to dressing), duration of mechanical ventilation, postoperative length of stay, the usage of vasoactive drugs and anesthetics, and fluid infusion and blood loss were also measured.

### Sample size

The sample size was calculated using GPower 3.1.lik. after the pre-experiment and before the formal experiment. The pre-experiment results showed a delirium incidence of 45% in the L group and 20% in the H group. This difference was assumed to be statistically significant. With two-sided α = 0.05 and 80% power, at least 52 patients were needed in each randomized group to test this difference in delirium incidence. A total of 116 patients (58 patients in each group) were eventually included in the study with a 10% drop-out rate.

### Statistical analysis

The statistical analysis included all randomized patients. Patients who were removed after randomization were excluded. Statistical analysis was done by a researcher who was proficient in the use of IBM SPSS Statistics 26. lnk. The Shapiro-Wilk test was used to test the normality of the quantitative data. The quantitative data conforming to a normal distribution were assessed between groups using two independent sample t-tests and presented as mean ± standard deviation. The quantitative data conforming to the skewed distribution and ranked data were assessed using a nonparametric test and described as median and interquartile range. The qualitative data between the two groups were assessed with χ^2^ test and presented as frequency and percentage. A *P* value < 0.05 was considered statistically significant. To assess associations between delirium and other variables, we used logistic regression to address potential bias.

## Results

**Fig 1:**
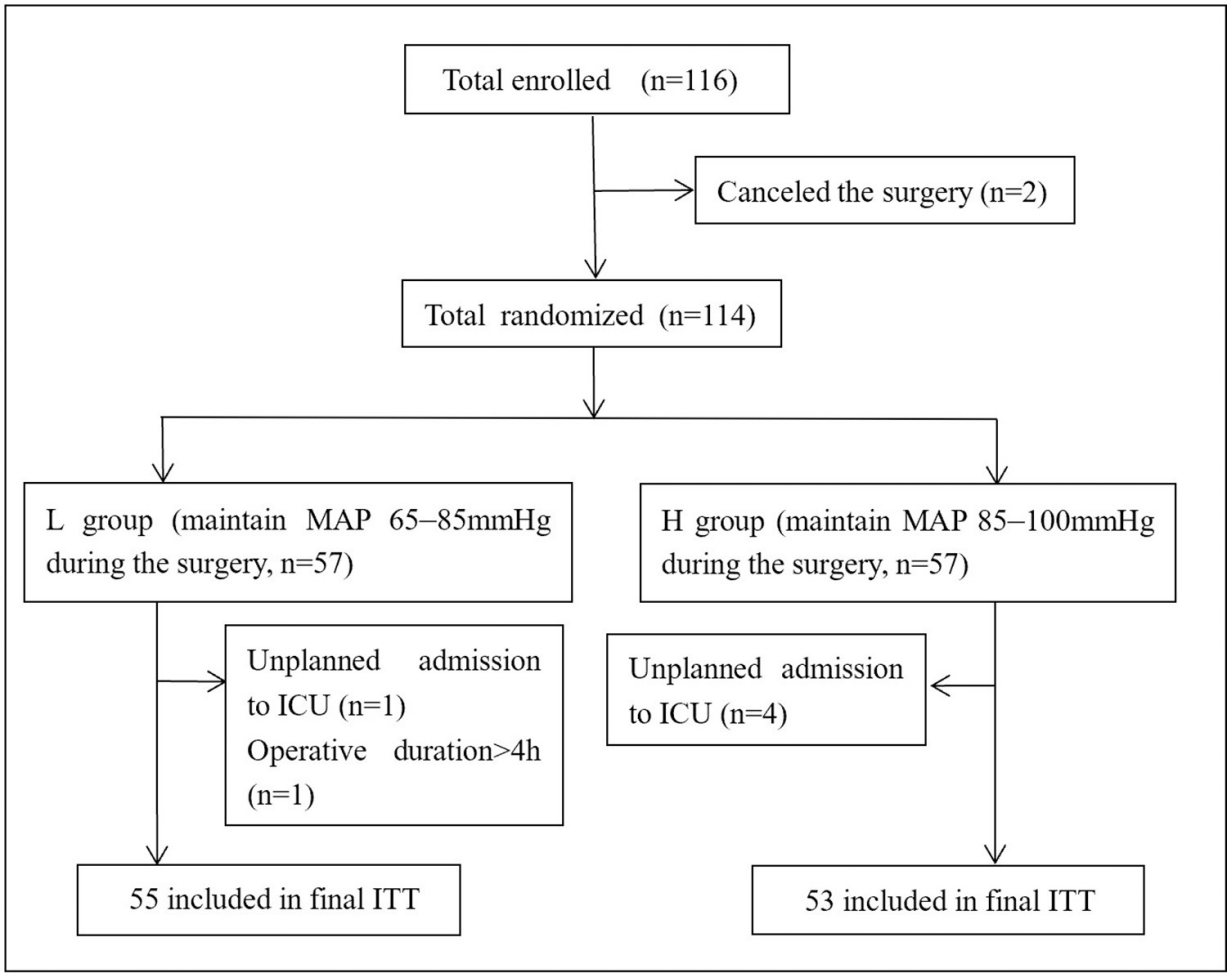
Trial profile. (ITT = intention-to-treat).

A total of 116 patients signed informed consent and were included in the study from April 2020 to August 2021, of whom 2 were excluded because the surgery was canceled unexpectedly and 114 patients were randomly assigned. Five patients were removed for unplanned admission to the ICU and one for operative duration longer than 4 h after receiving the study intervention and finally 108 (L group n = 55, H group n = 53) included in ITT (**Fig 1**). The baseline characteristics (sex, age, BMI, ASA status, educational background, MMSE score, preoperative hemoglobin and albumin levels, history of operation, hypertension, diabetes, and coronary heart disease) were listed in **Table 1**, few significant differences were observed between the two groups (*P* > 0.05).

**Table 1:** Baseline characteristics of study participants by treatment group.

Characterictics	All (n = 108)	L group (n = 55)	H group (n = 53)	*P* value
Sex, female, n(%)	37(34.3)	18(32.7)	19(35.8)	0.733
Age, median(Q1-Q3), yr	71(68–75.75)	70(68–75)	71(67.5–76)	0.791
BMI, mean±SD, kg/m^2^	24.1±2.8	24.1±2.6	24.2±3.0	0.846
ASA Ⅲ, n(%)	23(21.3)	11(20)	12(22.6)	0.737
Educational background n(%)				
Primary school	54(50.0)	28(50.9)	26(49.1)	0.846
Secondary school	27(25.0)	14(25.5)	12(22.6)	
University school	27(25.0)	13(23.6)	15(28.3)	
MMSE score, median(Q1-Q3)	27(24–29)	26(23–28)	27(24–29)	0.504
Preoperative hemoglobin, mean±SD, g/L	125.6±15.6	124.5±16.9	126.7±14.1	0.471
Preoperative albumin, mean±SD, g/L	38.9±4.3	38.6±3.8	39.2±4.8	0.436
Operation history, n(%)	37(34.3)	20(36.4)	17(32.1)	0.639
Hypertention, n(%)	46(42.6)	25(45.5)	21(39.6)	0.540
Diabetes, n(%)	21(19.4)	10(18.2)	11(20.8)	0.736
Coronary heart disease, n(%)	9(8.3)	5(9.1)	4(7.5)	1.000

Abbreviations:BMI, body mass index; ASA, American Society of Anesthesiologists; MMSE, Mini-mental State Examination; Q1, First Quartile; Q3, Third Quartile; SD, standard deviation.

**Fig 2:**
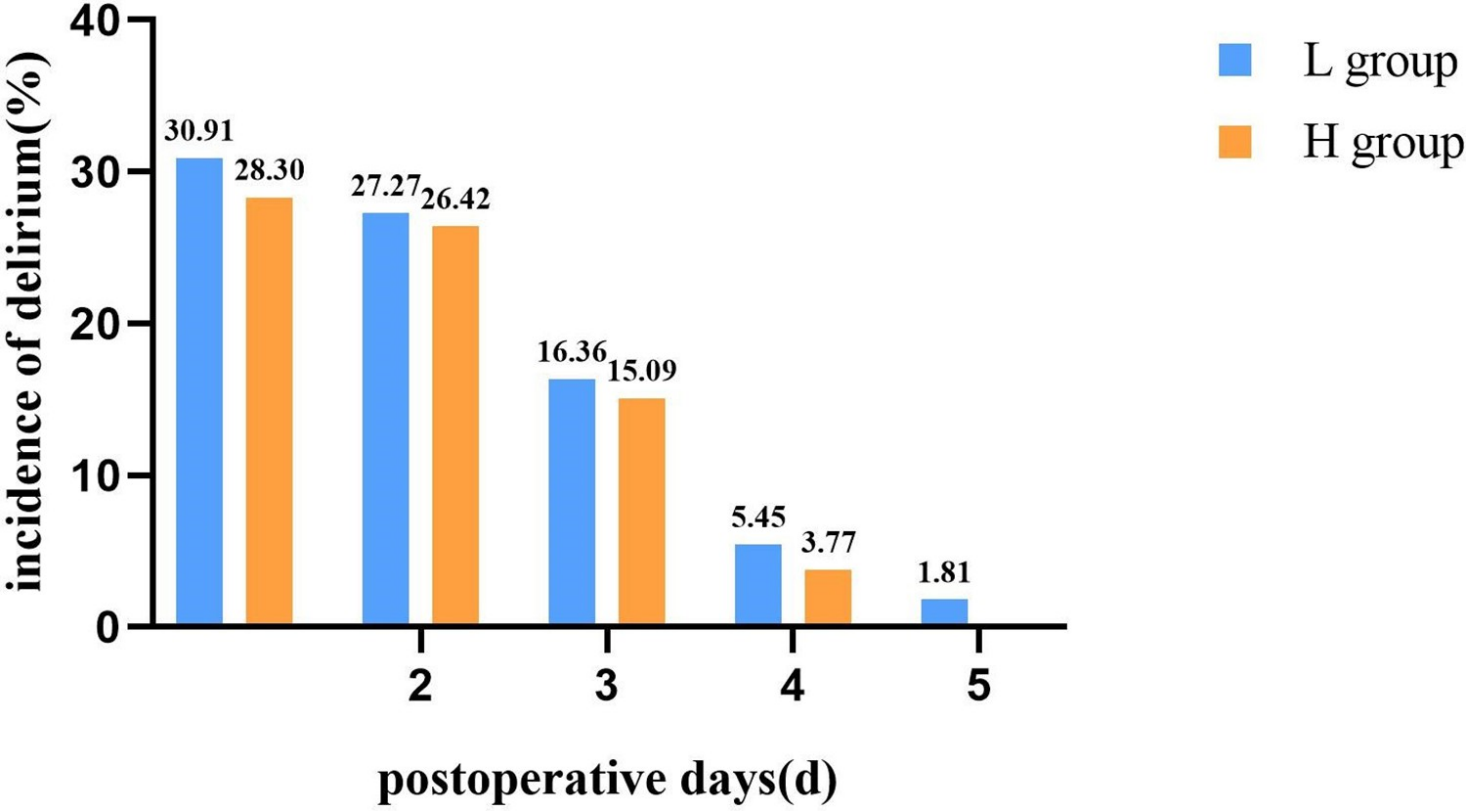
The incidence of delirium during the first 5 postoperative days.

Delirium occurred total in 33 (30.6%) out of 108 patients, among which 14 (13.0%) were hypoactive delirium, 6 (5.6%) were hyperactive delirium and 13 (12.0%) were mixed delirium. There were no significant differences in total delirium incidence (32.7% vs 28.3%, *P* = 0.618) and subtype distribution (hypoactive delirium 14.5% vs 11.3%, hyperactive delirium 7.3% vs 3.8%, mixed delirium 10.9% vs 13.2%, *P* = 0.730) between the two groups (**Table 2**).The incidence of delirium on the first postoperative day in the L group was 30.91% (17/55), the second day was 27.27% (15/55), the third day was 16.36% (9/55), the fourth day was 5.45% (3/55), the fifth day was 1.81% (1/55) and that in the H group respectively were 28.30% (15/53), 26.42% (14/53), 15.09% (8/53), 3.77% (2/53) and 0 (**Fig 2**). There was no difference between patients received intraoperative blood transfusion and patients who did not receive blood transfusion.

**Table 2:** The primary and secondary outcomes and surgery information.

	All (n = 108)	L group (n = 55)	H group (n = 53)	*P* value
Postoperative delirium, n(%)				
Total	33(30.6)	18(32.7)	15(28.3)	0.618
Hypoactive delirium	14(13.0)	8(14.5)	6(11.3)	0.730
Hyperactive delirium	6(5.6)	4(7.3)	2(3.8)	
Mixed delirium	13(12.0)	6(10.9)	7(13.2)	
D-O-M score, (Q1-Q3)	13(11.5–17)	14.5(12–18.5)	12(10–14)	0.022
Types of diagnosis, n(%)				
Malignant lesions	51(47.2)	27(49.1)	24(45.3)	0.692
Non-malignant lesions	57(52.8)	28(50.9)	29(54.7)	
Anesthesia duration, mean±SD, min	146.3±22.0	147.5±23.0	145.0±21.0	0.556
Operation duration, mean±SD, min	131.8±22.8	132.9±23.3	130.7±22.4	0.622
Duration of mechanical Ventilation, mean±SD, min	160.0±23.3	161.1±23.8	158.8±23.0	0.617
Postoperative length of stay, median(Q1-Q3),d	13(12–16)	13(12–16)	13(12–15.5)	0.877
Fluid infusion, median(Q1-Q3), ml	2000(1750–2500)	2000(1725–2500)	2250(1825–2500)	0.115
Blood loss, median(Q1-Q3), ml	100(80–200)	100(80–200)	100(80–190)	0.732
Incidence, n(%)				
Blood transfusion	16(14.8)	7(12.7)	9(17.0)	0.534
Atropine	30(27.8)	14(25.5)	16(11.3)	0.583
Esmolol	10(9.3)	6(10.9)	4(7.5)	0.787
Dosage, median(Q1-Q3)				
Propofol, mg	900(722.5–1050)	900(700–1000)	900(790–1100)	0.491
Sufentanil, μg	35(30–40)	35(30–40)	35(30–40)	0.749
Remifentanil, μg	825(700–1000)	800(660–1000)	900(700–1060)	0.379
Cisatracurium, mg	22(20–25)	21(19–25)	23(20–26)	0.331
Norepinephrine, μg	28(16–72)	20(14–40)	60(28–95)	0.001
Urapidil, mg	25(15–40)	25(20–50)	20(10–30)	0.001

Abbreviations: D-O-M, Delirium-O-Meter; Q1, First Quartile; Q3, Third Quartile; SD, standard deviation.

**Fig 3:**
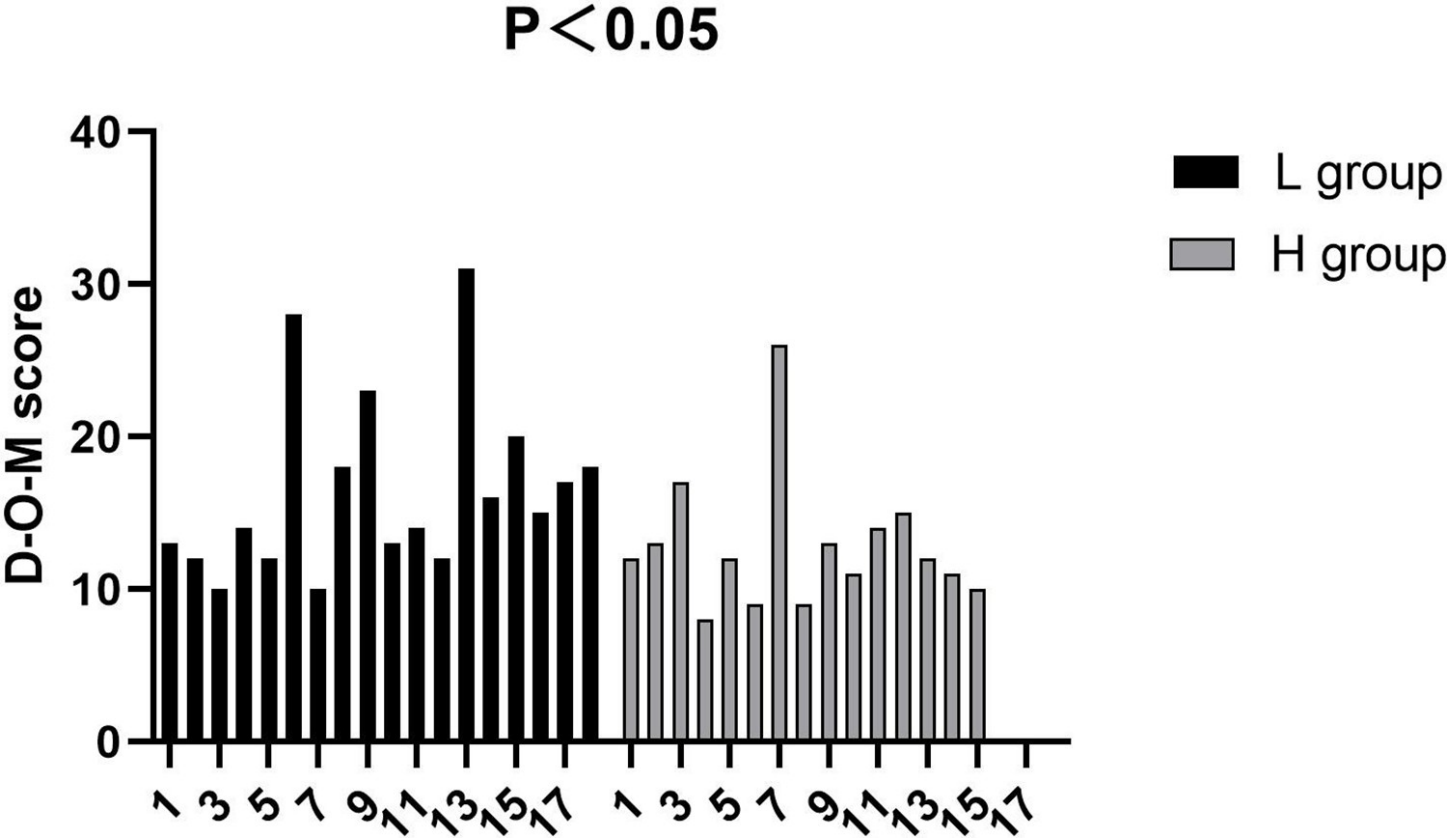
The D-O-M scores of the first episode of delirium of the two groups.

The median of D-O-M scores of the first episode of delirium of L group was 14.5 (quartile Q1 = 12, Q3 = 18.5) and that of the H group was 12 (Q1 = 10, Q3 = 14), (*P* = 0.022), (**Table 2** and **Fig 3**). The median norepinephrine usage of L group was 20 (Q1 = 14, Q3 = 40) mg and that of the H group was 60 (Q1 = 28, Q3 = 95) mg (*P <* 0.001). The median urapidil usage of the two groups were 25 (Q1 = 20, Q3 = 50) vs 20 (Q1 = 10, Q3 = 30) mg (*P <* 0.001). No significant differences were seen in the following variables between groups (*P* > 0.05): anesthesia duration, operation duration, duration of mechanical ventilation, postoperative length of stay, fluid infusion, blood loss, the usage of atropine, esmolol, propofol, sufentanil, remifentanil and cisatracurium (**Table 2**).

**Fig 4:**
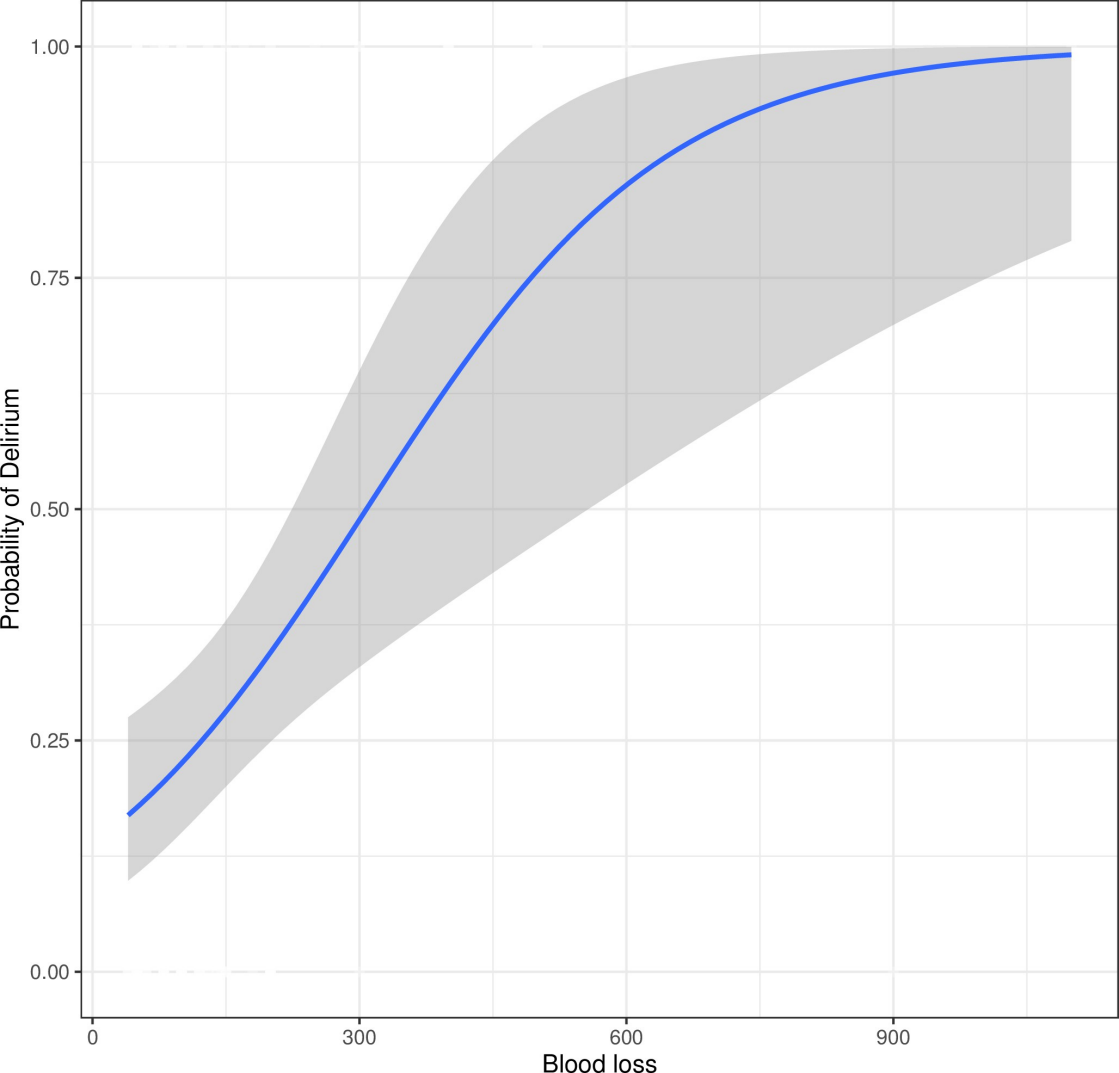
The association between blood loss and probability of delirium.

Logistic regression analysis revealed that postoperative length of stay (Odd Ratio [OR] = 1.107, 95%CI = 1.018–1.219, *P* = 0.024), fluid infusion (OR = 1.001, 95%CI = 1.000–1.001, *P* = 0.024) and blood loss (OR = 1.006, 95%CI = 1.003–1.010, *P* = 0.001) were associated with delirium in patients who received for gastrointestinal laparoscopic surgery. Multivariable setting was used to analyze the significance of three variables and found that only blood loss was associated with postoperative delirium (OR = 1.005, 95%CI = 1.002–1.010, *P* = 0.004) (**Table 3**). Logistic curve was used to show the association between blood loss and probability of delirium (**Fig 4**).

**Table 3:** Association between delirium and variables.

Terms	Crude OR (95%CI)	Uni-*P* value	Adj OR (95%CI)	multi-*P* value
Age	1.048(0.971–1.132)	0.228		
HB	0.996(0.969–1.022)	0.747		
Albumin	0.958(0.867–1.055)	0.288		
BMI	1.059(0.914–1.231)	0.450		
Anesthesia duration	1.008(0.990–1.027)	0.390		
Operation duration	1.009(0.990–1.027)	0.334		
Postoperative length of stay	1.107(1.018–1.219)	0.024	1.093(0.998–1.207)	0.062
Fluid infusion	1.001(1.000–1.001)	0.024	1.000 (0.999–1.001)	0.858
Blood loss	1.006(1.003–1.010)	0.001	1.005(1.002–1.010)	0.004

## Discussion

This study showed that maintaining intraoperative MAP at 86–100 mmHg did not reduce the incidence of postoperative delirium in elderly patients undergoing gastrointestinal laparoscopic surgery compared with those whose MAP was 65–85 mmHg.

The relationship between intraoperative blood pressure management and postoperative delirium has always been controversial. However, it is entirely not possible to reduce the incidence of delirium by optimizing blood pressure management with simple interventions [12]. Despite the lack of a uniform threshold, hypotension has long been considered a risk factor for delirium [13, 14]. One reason for this view may be that hypotension reduces cerebral blood perfusion. A retrospective study of 1083 patients found that intraoperative and postoperative hypotension (MAP < 65 mmHg) exposure is associated with delirium [8]. Similarly, another study confirmed that a severe drop in MAP (≤ 49 mmHg) is one of the risk factors for hyperactive delirium in adult patients with postcardiotomy and with extracorporeal membrane oxygenation support [15]. There is also a new study showing that lower diastolic pressure may be more closely associated with the occurrence of delirium. The incidence of delirium in patients with at least one episode of diastolic pressure < 50 mmHg during operation reached up to 81%, which is far higher than that in patients with no diastolic pressure < 50 mmHg [9].

Hirsch suggested that absolute hypotension (MAP < 50 mmHg) or relative hypotension (decreases by 40%) bore no special relationship to delirium as well as the duration of hypotension [16, 17]. Conversely, drastic fluctuations in intraoperative blood pressure, as some scholars insist, were significantly associated with delirium [18, 19]. Another view that needs to be further confirmed by major clinical trials is that the effect of blood pressure on delirium is chronic rather than acute, which means there is no connection between intraoperative blood pressure value and postoperative delirium [11].

Recently, the concept of optimal MAP (MAPopt), which is based on continuous monitoring and analysis of cerebrovascular autoregulation using near-infrared spectroscopy, has been introduced into clinical research. When patients had a near MAPopt level of blood pressure, the risk of delirium and poor outcome were minimized [20]. A study of 103 elderly patients with hip fracture patients may confirm the existence of MAPopt. That study showed that the closer the intraoperative MAP was to 80 mmHg, the lower the risk of patients experiencing postoperative delirium [21]. However, as previous studies have demonstrated, the brain has a powerful capacity of cerebrovascular autoregulation. A bold prediction could be made that MAPopt is a range rather than an exact value. Studies have shown that tight control of intraoperative blood pressure (systolic pressure > 80% baseline pressure or MAP > 75 mmHg) significantly reduced the incidence of delirium compared to standard care [22]. However, some proposed that targeting MAP greater than the lower critical threshold of cerebrovascular autoregulation could realize a remarkable reduction in the incidence of delirium [12, 23].

The results of this study showed that a higher intraoperative blood pressure had a positive effect on delirium severity, but the postoperative length of stay did not decrease as expected. Delirium severity and its associated factors are also the focus of research recently. Pain, inflammation, hypoxemia, and cerebral injury were believed to bring about more severe delirium and accelerate the development of psychiatric disorders [24]. There is also some research showing a strong correlation between educational background and delirium severity. Kolanowski et al. claimed that lower education levels were associated with more severe delirium, and there were also gender differences in this association [25]. The negative effect of increased co-morbidity on the severity of delirium was more pronounced in women than in men, which means female patients may correspondingly gain a greater body resistance to severe delirium through promoting physical fitness and improving cognitive reserve than their male counterparts. Yet, researchers believe that medical factors are more of a stimulant for developing severe delirium than sociodemographic or psychological factors [26]. Thus, it could be assumed that the optimization of perioperative medical care may effectively reduce the severity of delirium, which is consistent with the results of this study. Other studies, however, argue that primary prevention could only reduce the frequency and duration of postoperative delirium, it does not play a positive role in lessening the severity of delirium [27].

It has been suggested that intraoperative allogeneic transfusion is an independent risk factor for delirium and the risk had a dose-dependent effect with transfusion volume [28]. A study of hip fracture repair surgery also showed that delirium could be induced when blood transfusion exceeded two units [29]. Another current view is that having more than two transfusions is a risk factor for delirium [11]. Blood loss, well-known risk factors for delirium, might be risk factors for only hyperactive delirium [30]. This is consistent with our results showing that patients with higher blood loss is associated with postoperative delirium.

There were still some limitations of our study. First, this study did not assess long-term changes in cognitive function, which may be the direction of a future study. Second, patients with a history of hypertension were not excluded from the study, which may complicate the disturbance of potential confounders. Third, the evaluator of the incidence of delirium in this study was not an professional psychiatrist made the accuracy of the findings controversial to some extent. Therefore, further large sample multicenter studies are needed to confirm the relation between blood pressure and delirium.

## Conclusion

Maintaining intraoperative MAP at 86–100 mmHg did not reduce the incidence of postoperative delirium in elderly patients undergoing gastrointestinal laparoscopic surgery compared with those whose MAP was 65–85 mmHg. However, the severity of delirium could be reduced. In addition, intraoperative blood transfusions may increase the risk of delirium.

## Supporting information

S1 Checklist CONSORT 2010 checklist. (DOC)

S1 File CAM-CR (Confusion Assessment Method Chinese reversion). (PDF)

S2 File Study protocol (in Chinese). (PDF)

S3 File Study protocol (in English). (DOC)

S1 Data (XLSX)
