# Supplementary material for: Higher intraoperative mean arterial blood pressure does not reduce postoperative delirium in elderly patients following gastrointestinal surgery: A prospective randomized controlled trial
Source: PLoS One. 2022 Dec 22;17(12):e0278827. doi: 10.1371/journal.pone.0278827 (PMC9778934; doi:10.1371/journal.pone.0278827)
Supplement: S1 File — (PDF) [file pone.0278827.s002.pdf]

CAM-CR

（1） Acute onset: (Time from the previous drive to the development of the disease) Is there evidence of acute changes in the mental condition of patients?

1．absent

2．mild disturbances：Three days to seven days

3．moderate：One day to three days

4．severe：Within one day

（2）Inattention : (Please say all the odd numbers between 21 and 1 in order) Is it difficult for patients to concentrate?

1．absent

2．mild disturbances：1-2 errors

3．moderate：3-4 errors

4. severe：5 or more errors

（3）Mind chaos:Is the patient's thinking messy or discomfort? For example, the theme of the conversation is scattered or unwilling, the thinking is unclear or logical, or suddenly transferred from one topic to another topic?

1．absent

2．mild disturbances：Occasionally, the brief words are blurred or incomprehensible, but they can still talk smoothly

3．moderate：The short -term words are incomprehensible, and the conversation has a significant impact

4．severe： Words are incomprehensible most of the time, and it is difficult to make effective conversations

(4) Changes in the level of consciousness：

1．absent：Machinery (normal)

2．mild disturbances：Alertness (highly vigilant and excessive sensitivity to environmental stimuli)

3．moderate：Dissatious (sleepy, but easy to wake up) or sleepy (difficult to awaken)

4． severe： Comat (cannot be awakened)

(5) Direct disorders: Is there any directional disorder at any time at the meeting? For example, he believes that he is in other places rather than in a hospital, using the wrong bed, or wrong judgment of a day or wrong time or space orientation based on MMSE?
1． absent
2. mild disturbances：Occasionally there are targeted errors in time or place (close to correct), but it can be corrected by itself
3． moderate： Frequent orientation errors in time or place, but self -oriented
4． severe： Time, place and self -directional poor

(6) Memory loss (mainly based on the three words in MMSE)
Do patients show memory problems during the interview? For example, cannot recall what happened in the hospital, or it is difficult to recall instructions (including three words in MMSE)?
1． absent
2． mild disturbances： There is a word that cannot be recalled or memories errors
3． moderate： There are two words that cannot be recalled or memorable errors
4． severe： There are three words that cannot be recalled or memorable errors

(7) Perception: Does the patient have evidence of conscious obstacles? For example, hallucinations, illusions, or interpretations of things (for example, when something is not moved, and patients think it is moving)?
1. absent
2. mild disturbances:There are only auditory hallucination
3. moderate:There is an visual hallucination , accompanied by or not with auditory hallucination
4. severe:There is an tactile or olfactory or gustatory hallucination, accompanied by or not with auditory hallucination

(8) Spiritual excitement： During the interview, does the patient have an abnormal increase in behavioral activities? For example, sitting uneasily, tap fingers or suddenly change the position?
1. absent

2．mild disturbances：Occasionally restless, anxiety, tap finger and jitter
3．moderate：Walking around repeatedly, obvious excitement
4．severe：The behavior is chaotic and needs to be restrained

（**9**） Psychomotor retardation/hypokinesia: During the interview, does the patient have abnormalities in the level of exercise behavior? For example, often lazy, slowly enter a certain space, stay for a long time or move slowly?
1. absent
2．mild disturbances：Occasionally slower than previous activities, behaviors and movements
3．moderate：Always maintain a posture
4．severe：Stiff state

（**10**） Volatility: Does the patient's mental condition (attention, thinking, direction, memory) fluctuate before or in the interview?
1．absent
2．mild disturbances：Occasionally fluctuating in the day
3．moderate：Symptoms worsen at night
4．severe：Symptoms fluctuate violently during the day

（**11**） Sleep -Awakening cycle changes: (excessive sleep during the day and insomnia at night) Does the patient have evidence of sleep -awakening cycle disorders? For example, excessive sleep during day and night insomnia?
1．absent
2．mild disturbances：There are occasional dozing during the day, and wake up while sleeping at night
3. moderate：There are ofen dozing during the day, and wake up while sleeping at night or hard to fall asleep
4．severe：There are often fall asleep during the day and can't fall asleep at night

Note:
19 points below to prompt the patient to have no delirium
20-22 points suggest that the patient is suspicious of delirium
22 points and above, it is prompted that the patient is delirium
