# Supplementary material for: Higher intraoperative mean arterial blood pressure does not reduce postoperative delirium in elderly patients following gastrointestinal surgery: A prospective randomized controlled trial
Source: PLoS One. 2022 Dec 22;17(12):e0278827. doi: 10.1371/journal.pone.0278827 (PMC9778934; doi:10.1371/journal.pone.0278827)
Supplement: S2 File — (PDF) [file pone.0278827.s003.pdf]

徐州医科大学

研究生课题设计书

术中平均动脉压与术后谵妄：一项前瞻性随机对照临床研究

研究生学院 制

1. 本研究课题的科学依据和意义(国内外研究概况,水平和发展趋势,立题依据有何特色和创新之处)

术后谵妄是一种术后急性的精神紊乱状态，常伴有短暂性的注意力、感受、思维、记忆和睡眠周期障碍，其中注意力障碍是其核心症状。谵妄（delirium）一词源于拉丁语“ delirare”，虽然2500多年前就有人对谵妄进行过描述，但直到上世纪90年代中期，美国精神病学协会（APA）的《精神疾病统计手册》第四版（DMS－IV）才正式对谵妄进行了定义。到第五版DSM－Ⅴ则对谵妄诊断标准更为明确，主要集中在注意障碍及认知功能障碍两种临床症状，强调了不能用其他先前存在的、已经确立的或正在进行的神经认知障碍来更好地解释，也不是出现在觉醒水平严重降低的背景下，并要求根据不同病因及病程来对谵妄进行描述（物质中毒性谵妄、物质戒断性谵妄、药物所致谵妄、由于其他躯体疾病所致的谵妄、由于多种原因所致的谵妄等在内的七种谵妄）（急性或慢性）。

在老年住院患者中谵妄的发生率一直居高不下。在老年髋部骨折手术后，术后谵妄的发生率可高达30%左右[1, 2]，在心脏手术患者中甚至更高。根据临床表现不同，可将谵妄分为三个亚型：躁狂型、抑郁型和混合型，其中发生率最高的是抑郁型，但也最容易被忽视，往往也预示着更坏的预后，混合型次之，躁狂型的发生率最低。但是无论何种谵妄，都会降低患者的生活质量，增加患者及其家属的心理和经济负担。

谵妄的病因多且不明确，谵妄病因学的多因素模型已得到充分验证和广泛接受。痴呆、高龄和疾病等都是谵妄发生的共同危险因素，另外还有非常多的诱发因素，包括急性疾病、外科手术、创伤和药物等等。从病理生理的机制来看，老年患者之所以更容易发生谵妄，是由于高龄常伴有脑器质性病变造成大脑储备下降、代谢水平降低、视觉与听觉障碍导致知觉减退、神经递质合成减少（如乙酰胆碱）、与年龄有关的药物动力学和药效学的改变、内稳态调节机制的减弱等所致。在老年人中，神经元、树突、受体和小胶质细胞的永久性损伤逐渐积累，以及脑血管疾病或头部创伤的影响，可能使老年人，特别是那些潜在的认知障碍患者，在受到生理压力时更容易发生谵妄[3, 4]。

目前尚无治疗术后谵妄的特异性方法和药物，因此重点在于预防。由于可能诱发谵妄的因素很多，故需在术前、术中和术后的各个环节上加以注意，积极纠正危险因素，尽量减少术后谵妄的发生。其中术中干预措施中和麻醉关系密切的主要的干预措施包括维持充足的氧供、合适的血压和血红蛋白水平、水电解质平衡和正确用药。麻醉方法应尽可能简单由于老年人对药物反应敏感，而且代谢或肾功能异常，也使药物的半衰期延长，所以麻醉用药要谨慎，尽量不用或少用中枢性抗胆碱能药物（如阿托品、东莨菪碱）。

有研究表明术中维持血压在较低或较高水平都不利于降低谵妄的发生率[5, 6]，但也有研究表明高血压和低血压与谵妄的发生均无显著相关性，但是术中血压的波动却可以显著增加谵妄的发生率[7, 8]。血压与术后谵妄的关系尚不明确，因此本研究拟选择择期行胃肠手术的老年患者作为

实验对象用以探究术中血压调控水平对术后谵妄的影响，为科学的认识和预防谵妄的发生提供依据。

2. 参考文献

[1] Wang et al.Incidence and risk factors of postoperative delirium in the elderly patients with hip fracture[J].*Journal of Orthopaedic Surgery and Research.* 2018; 13:186

[2] Furlaneto ME, Garcez-Leme LE. Delirium in elderly individuals with hip fracture: causes, incidence, prevalence, and risk factors[J]. *Clinics*. 2006; 61(1):35-40.

[3] Rudolph JL, Marcantonio ER. Review articles: postoperative delirium: acute change with long-term implications. *Anesth Analg*. 2011; 112: 1202–11.

[4] Sugimura Y, Sipahi NF, Mehdiani A, et al. Risk and Consequences of Postoperative Delirium in Cardiac Surgery. *Thorac Cardiovasc Surg.* 2020; 68: 417–424.

[5] Maheshwari K, Ahuja S, Khanna AK, et al. Association Between Perioperative Hypotension and Delirium in Postoperative Critically Ill Patients: A Retrospective Cohort Analysis[J]. *Anesth Analg*. 2020; 130(3): 636-643.

[6] Wang H, Hou D, Tian X, et al. Risk factors for agitation and hyperactive delirium in adult postcardiotomy patients with extracorporeal membrane oxygenation support: an observational study[J]. *Perfusion*. 2020; 35(6): 534-542.

[7] J. Hirsch1, G. DePalma al.Impact of intraoperative hypotension and blood pressure flfluctuations on early postoperative delirium after non-cardiac surgery†[J].*British Journal of Anaesthesia*.2015.

[8] Citation: Wang N-Y, Hirao A, Sieber F (2015) Association between Intraoperative Blood Pressure and Postoperative Delirium in Elderly Hip Fracture Patients. *PLoS ONE.* 10(4): e0123892.

3. 研究内容和预期结果(说明课题的具体研究内容、重点解决的科学技术问题、预期成果提供形式)

研究内容：

本研究拟选择择期行胃肠手术的老年患者作为实验对象用以探究术中血压调控水平对术中脑氧饱和度和术后谵妄的影响，为科学的认识和预防谵妄的发生提供依据。该研究为随机对照临床试验，采用随机数字法将所有符合入选标准且不符合任何排除标准的患者按照 1：1 分为低压组和高压组，低压组患者术中维持 65mmHg≤MAP≤85mmHg, 高压组患者术中维持 86mmHg≤MAP≤100mmHg, 直到手术结束后 5min。

重点解决的科学技术问题：

(1) 探究术中血压维持水平与术后谵妄的关系。

(2) 为科学的认识和预防谵妄的发生提供依据。

预期成果提供形式：

通过术中维持血压在不同水平用以探究血压与术后谵妄的关系，预防老年患者术后谵妄的发生，提高其预后。预期国内交流 1 次，发表 1-2 篇文章。提高临床医生对术后谵妄的科学认识。

4. 拟采取的研究方法和实施方案(包括科研方法、步骤、主要技术指标、资料的统计学处理和可能遇到的问题及解决办法)

研究方法和技术路线：

纳入标准：患者必须满足以下所有入选标准才可入组本研究

1）65 岁≤年龄≤85 岁，性别不限；

2）行择期腹腔镜下胃肠手术而无计划入住 ICU；

3）ASA 评分为Ⅱ级或Ⅲ级；

4)清楚了解、自愿参加该项研究，并由本人签署知情同意书；

排除标准：具有以下任何一项的患者不能入组本研究

1）脑血管意外史；

2）高血压病史；

3）入院前半年内服用过精神类药物史；

4）既往有精神分裂或癫痫、老年痴呆；

5）颈部超声显示颈内血管有明显斑块；

6）视觉、听觉或语言交流障碍；

7）长期酗酒；

8）急诊手术；

9)术前进行简易智力状态量表（MMSE）评分不能配合及低于相应文化程度最低评分（文盲≤17 分，小学≤20 分，中学≤22 分，大学≤23 分）；

10)研究者认为不宜参加此试验的其他情况；

剔除标准：

1）血压水平在目标范围内时间占总手术时间小于 80%

2）非计划入住 ICU

3）手术时间＞4h 或者＜1h

4）术后不配合谵妄评估

术前常规禁食 8 h，禁饮 4 h,入室后常规监测无创血压、ECG 和 SpO_2 。开放上肢静脉，行桡动脉穿刺置管监测动脉血压。用酒精棉球消毒患者前额皮肤后，将脑氧饱和度监测仪探头置于患者眉弓上方，并将探头完全避光，使用红外光谱测量 rSO_2 。

麻醉诱导：依此缓慢静脉注射舒芬太尼 0.3～0.5μg／kg、丙泊酚 1～2mg／kg、顺式阿曲库铵 0.2 mg／kg，预充氧后气管插管接麻醉机行机械通气，FiO_2 60％，VT 6～8 ml／kg，RR 12～15 次／分，I：E 1：2，$PETCO_2$ 35～45 mmHg。全凭静脉维持麻醉：丙泊酚 4 ～8 $mg \cdot kg^{-1} \cdot h^{-1}$、瑞芬太尼 0.1～0.3 $\mu g \cdot kg^{-1} \cdot min^{-1}$、顺式阿曲库铵 0.1～0.2 $mg \cdot kg^{-1} \cdot h^{-1}$，维持 BIS 值

在 40 ～ 60。手术结束后清醒拔管转入PACU,Aldrete评分达10分时转入病房，离室前留取术后动脉血标本

血压调控方案：

1）调节有效循环血容量：加快或减慢输液速度，晶体液、胶体液、红细胞、血浆

2）调节血管张力：去甲肾上腺素、乌拉地尔

3）调节心率：HR＜50次/分，阿托品，HR＞100次/分，艾司洛尔

主要测量指标：

1）术后5天内谵妄发生率

次要测量目标：

1）术中脑氧饱和度

2) 手术时间和机械通气时间

3) 术中麻醉药物、血管活性药物使用量

4）PACU滞留时间

5）液体输入量和失血量

技术路线图:

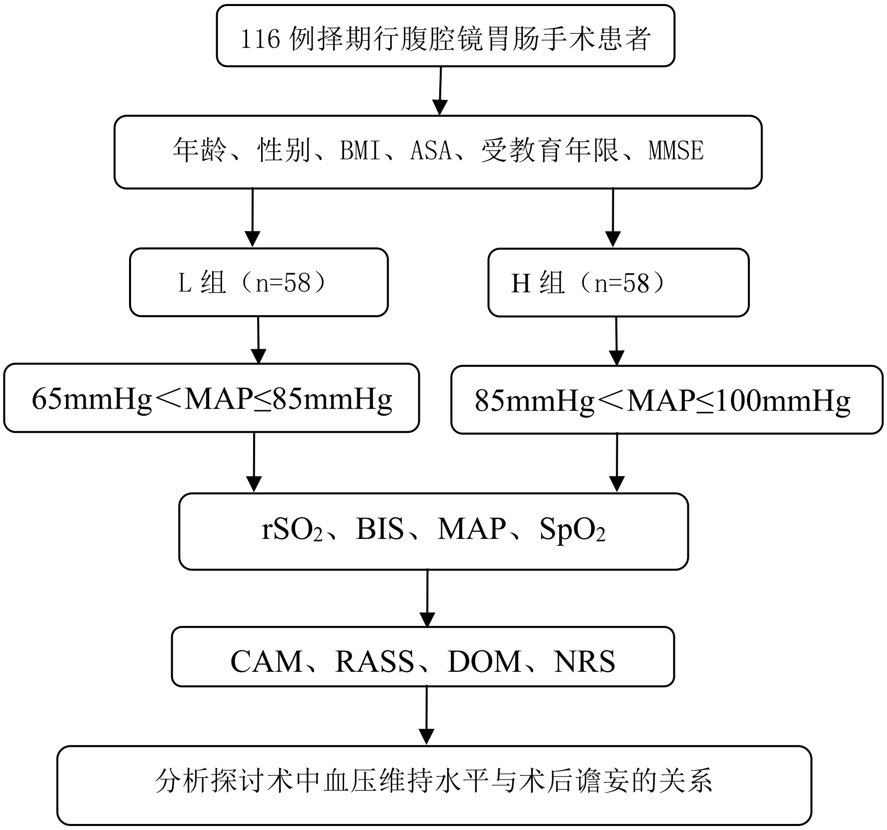

统计分析：

所有数据的统计分析均使用 IBM SPSS Statistics 26. lnk 统计软件完成。数据的正态性检验使用 S-W 检验。符合正态分布的定量资料组间比较使用两独立样本 t 检验，组内比较使用成对样本 t 检验，结果以均数±标准差（ $\bar{x} \pm s$）表示。符合偏态分布的定量资料和等级资料使用非参数检验，结果以中位数和四分位间距表示。定性资料组间比较使用χ^2检验，结果以例（%）表示。$P < 0.05$ 为差异有统计学意义。

问题：患者对实验表示不理解，不愿意配合。

解决方法：患者被告知主要实验方法及可能发生的不良反应，签署知情同意书，保障患者的知情同意权。不同意签署知情同意书者则纳入排除标准。

5. 实现本课题已具备的条件(开展本课题的技术优势、现有仪器设备、技术人员及协作条件,以及预试验情况)

1）项目研究的基础，技术优势和仪器设备；

本科室大量的临床病例资料等为本课题的顺利完成做了坚实的保证，本科室每年完成胃肠手术约为 500 例，可在规定时间内完成临床相关试验。拥有脑氧检测仪等设备。

2）项目研究具备的人才队伍条件；

本课题组各成员大部分为硕士研究生或正在攻读博士的研究生，研究生期间受过良好的科研训练，在读研期间参与过国家自然科学基金资助项目、江苏省卫生厅、科技厅等课题，均积累了一定的实验相关理论方面的基础。同时，本课题组成员另有高级职称人员 3 名，中级 3 名，人员配备合理，不仅具有高年资高级职称医师的指导，而且中初级职称医师配合进行临床试验。因此，能够保质保量的完成研究的工作。

3）项目承担单位资金配套能力及科技服务管理能力；

连云港市第一人民医院大力支持员工申报课题，并支持员工创新，为项目提供优质的试验环境，科学的服务管理能力。

6.以往研究成果、获奖、论文、专利（名称、颁发单位、排名、时间、编号）

（1）Pin Zhu, Xiaobao Zhang, Hengfei Luan, Jiying Feng, Jizheng Cui, Yong Wu, **Zhibin Zhao（赵志斌）**,* Ultrasonographic measurement of the subclavian vein diameter for assessment of intravascular volume status in patients undergoing gastrointestinal surgery: comparison with central venous pressure. J Surg Res, 2015, 196:102-106.

（2）Zhang XB, Luan HF, Zhu P, Feng JY, Cui JZ, **Zhao ZB（赵志斌）***. Does ultrasonographic measurement of the inferior vena cava diameter correlate with central venous pressure in the assessment of intravascular volume in patients undergoing gastrointestinal surgery?J Surg Res.2014 Oct;191(2):339-43.

（3）Zhang XB, Feng JY, Zhu P, Luan HF, Wu Y, **Zhao ZB（赵志斌）***. Ultrasonographic measurements of the inferior vena cava variation as a predictor of fluid responsiveness in patients undergoing anesthesia for surgery. J Surg Res.. 2016,204(1):118-122.

（4）杨茜茜，**赵志斌 ***，等.趋化因子 CX3CL1 和受体 CX3CR1 在中枢神经系统疾病中的进展.医学研究生学报.2020，33（05）：416-421.
