# Supplementary material for: Higher intraoperative mean arterial blood pressure does not reduce postoperative delirium in elderly patients following gastrointestinal surgery: A prospective randomized controlled trial
Source: PLoS One. 2022 Dec 22;17(12):e0278827. doi: 10.1371/journal.pone.0278827 (PMC9778934; doi:10.1371/journal.pone.0278827)
Supplement: S3 File — (DOC) [file pone.0278827.s004.doc]

**Xuzhou Medical University**

**Postgraduate project design book**

**Intraoperative mean arterial blood pressure and postoperative delirium: a prospective controlled clinical trial**

**Graduate school system**

1. The scientific basis and significance of this research topic (research overview, level and development trend at home and abroad, what are the characteristics and innovations of the basis for the establishment of the topic)

Postoperative delirium is an acute post-operative mental disorder, often accompanied by transient disturbances in attention, feeling, thinking, memory, and sleep cycles, of which attention disturbance is its core symptom. The term delirium is derived from the Latin "delirare", and although delirium has been described more than 2,500 years ago, it was not until the mid-1990s that delirium was formally defined in the DMS-IV. In DSM-V, the diagnostic criteria for delirium are more clear, mainly focusing on two clinical symptoms of attention deficit and cognitive dysfunction, emphasizing that other pre-existing, established or ongoing neurocognitive cannot be used, not in the context of severely reduced levels of arousal, and require a description of delirium according to the various etiologies and course of the disease (substance-toxic delirium, substance-withdrawal delirium, drug-induced delirium, delirium due to other Delirium due to physical disease, delirium due to multiple causes, etc.) (acute or chronic).

The incidence of delirium among elderly hospitalized patients has remained high. After hip fracture surgery in the elderly, the incidence of postoperative delirium can be as high as about 30% ^[1, 2]^. According to different clinical manifestations, delirium can be divided into three subtypes: hypoactive delirium, hyperactive delirium, and mixed delirium, of which hypoactive delirium is the most common, but it is also the most easily overlooked, and often indicates a worse prognosis. The mixed type is second, and the hyperactive type has the lowest incidence. However, no matter what kind of delirium, it will reduce the quality of life of patients and increase the psychological and economic burden of patients and their families^[4, 5]^.

The etiology of delirium is numerous and unclear, and multifactorial models of the etiology of delirium are well-validated and widely accepted. Dementia, advanced age, and disease are all common risk factors for delirium, and there are many other predisposing factors, including acute illness, surgery, trauma, and drugs. From the perspective of the pathophysiological mechanism, the reason why elderly patients are more prone to delirium is that old age is often accompanied by organic brain lesions, resulting in decreased brain reserves, decreased metabolic levels, visual and auditory disorders leading to decreased perception, and decreased neurotransmitter synthesis(such as acetylcholine), age-related changes in pharmacokinetics and pharmacodynamics, and weakened homeostasis regulatory mechanisms. The progressive accumulation of permanent damage to neurons, dendrites, receptors, and microglia in older adults, as well as the effects of cerebrovascular disease or head trauma, may predispose older adults to delirium when under physiological stress, especially those with underlying cognitive impairment.

There are currently no specific treatments and drugs for postoperative delirium, so the focus is on prevention. Because there are many factors that may induce delirium, it is necessary to pay attention to all aspects of preoperative, intraoperative and postoperative, actively correct risk factors, and minimize the occurrence of postoperative delirium. Among the intraoperative interventions, the main interventions closely related to anesthesia include maintaining adequate oxygen supply, appropriate blood pressure and hemoglobin levels, water and electrolyte balance, and correct medication. The method of anesthesia should be as simple as possible. Because the elderly are sensitive to drugs, and metabolic or renal dysfunction also prolongs the half-life of drugs, anesthetic drugs should be used with caution, and central anticholinergic drugs (such as atropine, scopolamine).

Some studies have shown that maintaining blood pressure at a lower or higher level during surgery is not conducive to reducing the incidence of delirium ^[5,6]^, but there are also studies showing that neither hypertension nor hypotension is significantly correlated with the occurrence of delirium, but Intraoperative blood pressure fluctuations can significantly increase the incidence of delirium^[7, 8]^. The relationship between blood pressure and postoperative delirium is still unclear. Therefore, this study intends to select elderly patients undergoing elective gastrointestinal surgery as the experimental subjects to explore the effect of intraoperative blood pressure regulation on postoperative delirium.

2. references

[1] Wang et al.Incidence and risk factors of postoperative delirium in the elderly patients with hip fracture[J].*Journal of Orthopaedic Surgery and Research.* 2018; 13:186

[2] Furlaneto ME, Garcez-Leme LE. Delirium in elderly individuals with hip fracture: causes, incidence, prevalence, and risk factors[J]. *Clinics*. 2006; 61(1):35-40.

[3] Rudolph JL, Marcantonio ER. Review articles: postoperative delirium: acute change with long-term implications. *Anesth Analg*. 2011; 112: 1202–11.

[4] Sugimura Y, Sipahi NF, Mehdiani A, et al. Risk and Consequences of Postoperative Delirium in Cardiac Surgery. *Thorac Cardiovasc Surg.* 2020; 68: 417–424.

[5] Maheshwari K, Ahuja S, Khanna AK, et al. Association Between Perioperative Hypotension and Delirium in Postoperative Critically Ill Patients: A Retrospective Cohort Analysis[J]. *Anesth Analg*. 2020; 130(3): 636-643.

[6] Wang H, Hou D, Tian X, et al. Risk factors for agitation and hyperactive delirium in adult postcardiotomy patients with extracorporeal membrane oxygenation support: an observational study[J]. *Perfusion*. 2020; 35(6): 534-542.

[7] J. Hirsch1, G. DePalma al.Impact of intraoperative hypotension and blood pressure

flfluctuations on early postoperative delirium after non-cardiac surgery†[J].*British Journal of Anaesthesia*.2015.

[8] Citation: Wang N-Y, Hirao A, Sieber F (2015) Association between Intraoperative Blood Pressure and Postoperative Delirium in Elderly Hip Fracture Patients. *PLoS ONE.* 10(4): e0123892.

3. Research content and expected results

research content:

This prospective controlled clinical trial enrolled 116 patients aged 65–85,who underwent gastrointestinal laparoscopic surgery at The Affiliated Lianyungang Hospital of Xuzhou Medical University. These patients were randomized 1:1 to a MAP goal of 65–85 mmHg (L group) or an 86–100 mmHg (H group). The primary endpoint was the incidence of postoperative delirium, assessed twice daily with the Confusion Assessment Method (CAM) and Richmond Agitation–Sedation Scale (RASS) during the first 5 postoperative days. Delirium severity was evaluated with the Delirium-O-Meter (D-O-M).

Key scientific and technological problems to be solved:

(1)To explore the relationship between intraoperative blood pressure maintenance and postoperative delirium.

(2)Provide a basis for scientific understanding and prevention of delirium.

Expected results :

By maintaining blood pressure at different levels during surgery to explore the relationship between blood pressure and postoperative delirium, prevent the occurrence of postoperative delirium in elderly patients and improve their prognosis. It is expected to have 1 domestic exchange and publish 1-2 articles. Improve clinicians' scientific understanding of postoperative delirium.

4. Research methods and implementation plans to be adopted (including scientific research methods, steps, main technical indicators, statistical processing of data, and possible problems and solutions)

Research methods and technical routes:

Patients were enrolled in the study if all the following criterion were met:

1. be due to undergo gastrointestinal laparoscopic surgery and unplanned to be admitted to ICU
2. aged between 65 and 85
3. 18kg/m2≦BMI≦30kg/m2

Patients were excluded if any of the following was present:

1. with history of cerebrovascular accident
2. with hypertension and have poor blood pressure control (systolic blood pressure exceeds 150 mmHg or diastolic blood pressure exceeds 90 mmHg)
3. with history of taking psychotropic drugs within half a year before hospitalization
4. diagnosed with schizophrenia, epilepsy or Alzheimer's disease
5. neck ultrasound shows plaques in the blood vessels
6. with visual, auditory, or language communication impairment
7. with history of drug or alcohol abuse within 1 year
8. had emergency surgery
9. with a score of Mini-mental State Examination (MMSE) lower than the minimum score for the corresponding education level (illiteracy<17, primary school<20, secondary school<22, university school<23)

Patient were removed from the trial if any of the following occurs:

1. the duration of meeting the MAP target wasless than 80% of the operative duration,
2. the operative duration was less than 1 h or more than 4 h,
3. unplanned admission to the ICU.

Routinely fasted for 8 hours and abstained from drinking for 4 h before surgery, and routinely monitored non-invasive blood pressure, ECG and SpO_2_ after entering the room. The upper extremity vein was opened, and the radial artery puncture was performed to monitor the arterial blood pressure. After disinfecting the patient's forehead skin with an alcohol swab, place the cerebral oxygen saturation monitor probe above the patient's brow arch, completely shield the probe from light, and measure rSO_2_ using infrared spectroscopy.

The induction of anesthesia was accomplished by intravenous administration of 0.3–0.5 μg/kg sufentanil, 1–2 mg/kg propofol, and 2 mg/kg cis-atracurium. The following were also administered intravenously: 0.1–0.3 μg/(kg·min) remifentanil, 4–8 mg/(kg·h) propofol, and 0.1–0.2 mg/(kg·h) cis-atracurium to maintain an appropriate anesthesia depth, which was monitored by bispectral index (BIS) to achieve a BIS value of 40–60. All patients were ventilated in volume-controlled mode with a 60% fractional concentration of inspired oxygen and tidal volume and respiratory rate were adjusted to achieve the partial pressure of end-tidal carbon dioxide fluctuating between 35 and 45 mmHg.

Intraoperative blood pressure was controlled within the target range from the following three aspects: circulatory volume, heart rate, and angiotasis. Fluid and blood products were replenished to maintain adequate circulation volume based on the duration of fasting and intraoperative blood loss. Heart rate was raised with atropine and lowered with esmolol based on the clinical experience of the anesthesiologist. If necessary, norepinephrine and urapidil were used to keep MAP in line with the blood pressure goals.

primary endpoint:

the incidence of postoperative delirium during the first 5 postoperative days

secondary outcomes:

1. Intraoperative cerebral oxygen saturation
2. duration of operation (from skin incision to dressing), duration of mechanical ventilation and postoperative length of stay
3. the usage of vasoactive drugs and anesthetics
4. fluid infusion and blood loss

Technical route:

116 patients

Age, Gender, BMI, ASA, Education, MMSE

L group ( *n*=58)

H group ( *n*=58)

65mmHg＜MAP≤85mmHg

85mmHg＜MAP≤100mmHg

rSO2、BIS、MAP、SpO2

CAM、RASS、DOM、NRS

To analyze the relationship between intraoperative blood pressure maintenance level and postoperative delirium

Statistical analysis

The Shapiro-Wilk test was used to test the normality of the quantitative data. The quantitative data conforming to a normal distribution were assessed between groups using two independent sample t-tests and presented as mean ± standard deviation. The quantitative data conforming to the skewed distribution and ranked data were assessed using a nonparametric test and described as median and interquartile range. The qualitative data between the two groups were assessed with χ^2^ test and presented as frequency and percentage. A *P* value < 0.05 was considered statistically significant.

Question: The patient expressed incomprehension to the experiment and was unwilling to cooperate.

Solution: The patient was informed of the main experimental methods and possible adverse reactions, and signed the informed consent form to protect the patient's right to informed consent. Those who did not agree to sign the informed consent form were excluded.

1. Technical advantages of this project, existing instruments and equipment, technical personnel and cooperation conditions
2. The basis of project research, technical advantages and equipment

The large number of clinical cases in the department has made a solid guarantee for the successful completion of this project. The department completes about 500 cases of gastrointestinal surgery every year, and can complete clinical related trials within the specified time. The department equipped with cerebral oxygen monitor and other equipment.

(2) Excellent research team for the project

Most of the members of this research group are postgraduates or PhD student. They have received good scientific research training during their postgraduate studies. There are 3 senior professional titles and 3 intermediate staff members in this research group. Therefore, the research can be completed with high quality.

Past research results:

（1）Pin Zhu, Xiaobao Zhang, Hengfei Luan, Jiying Feng, Jizheng Cui, Yong Wu, Zhibin Zhao* . Ultrasonographic measurement of the subclavian vein diameter for assessment of intravascular volume status in patients undergoing gastrointestinal surgery: comparison with central venous pressure. J Surg Res, 2015, 196:102-106.

（2）Zhang XB, Luan HF, Zhu P, Feng JY, Cui JZ, Zhao ZB*. Does ultrasonographic measurement of the inferior vena cava diameter correlate with central venous pressure in the assessment of intravascular volume in patients undergoing gastrointestinal surgery?J Surg Res.2014 Oct;191(2):339-43.

（3）Zhang XB, Feng JY, Zhu P, Luan HF, Wu Y, Zhao ZB*. Ultrasonographic measurements of the inferior vena cava variation as a predictor of fluid responsiveness in patients undergoing anesthesia for surgery.J Surg Res.. 2016,204(1):118-122.
